# Postmortem computed tomography assessment of intracardiac and intravascular blood clots and gravitational sedimentation: clinical and laboratory associations in 114 in-hospital deaths

**DOI:** 10.1007/s11604-025-01797-3

**Published:** 2025-05-25

**Authors:** Masanori Ishida, Akira Katayama, Go Shirota, Naomasa Okimoto, Hiroyuki Abe, Keisuke Nyunoya, Kotaro Fujimoto, Mariko Kurokawa, Masumi Takahashi-Mizuki, Shohei Inui, Shunichiro Orihara, Kazuhiro Saito, Tetsuo Ushiku, Osamu Abe, Wataru Gonoi

**Affiliations:** 1 Department of Radiology, School of Medicine, Tokyo Medical University, 6-7-1 Nishi-shinjuku, Shinjuku-ku, Tokyo, 160-0023 Japan; 2 Department of Health Data Science, School of Medicine, Tokyo Medical University, 6-1-1 Shinjuku, Shinjuku-ku, Tokyo, 160-8402 Japan; 3 Department of Radiology, Graduate School of Medicine, The University of Tokyo, 7-3-1 Hongo, Bunkyo-ku, Tokyo, 113-8655 Japan; 4 Department of Pathology, Graduate School of Medicine, The University of Tokyo, 7-3-1 Hongo, Bunkyo-ku, Tokyo, 113-8655 Japan

**Keywords:** Coagulation, Computed tomography, Forensic radiology, Hypostasis, Postmortem CT, Sedimentation

## Abstract

**Purpose:**

Postmortem computed tomography (PMCT) typically reveals blood clots and sedimentation in cardiac and vascular structures. We examined the associations between these postmortem findings and antemortem clinical and laboratory parameters in in-hospital death.

**Material and methods:**

This prospective study included 114 non-traumatic in-hospital deaths where PMCT was performed within 24 h postmortem. Two radiologists evaluated high-density areas in the right and left atria, pulmonary artery, and thoracic aorta, and classified them as blood clots or gravitational sedimentation. The clinical and laboratory data from the week before death were analyzed using univariate and multivariate logistic regression.

**Results:**

Interobserver agreement was excellent for all anatomical sites (κ = 0.87–0.91). Blood clot or blood sedimentation were observed in 34–53% of cases across different locations. Per univariate analysis, non-pneumonic infections, positive blood cultures, and elevated coagulation parameters (prothrombin time-international normalized ratio, activated partial thromboplastin time) were associated with gravitational sedimentation. In contrast, solid malignancies and higher values of hematologic parameters (platelet count, red blood cells, hemoglobin, neutrophil percentage) were associated with blood clot formation (all *p* < .05). Per multivariate analysis, non-pneumonic infections maintained strong associations with gravitational sedimentation across all sites (*p* < .05), while higher platelet counts independently predicted blood clot formation in the right atrium, left atrium, and thoracic aorta (*p* < .05).

**Conclusion:**

Postmortem gravitational sedimentation was associated with non-pneumonic infections, whereas clot formation correlated with higher platelet counts. These findings provide objective criteria for interpreting PMCT findings and may aid in evaluating patients’ antemortem clinical status, particularly when clinical information is limited.

## Introduction

Postmortem computed tomography (PMCT) commonly depicts intracardiac/intravascular changes, particularly blood clots and sedimentation in the heart and great vessels [1, 2]. Blood clots, corresponding to "chicken fat clots" traditionally observed at autopsies, appear as high-density, cast-like structures within the vessels. Blood sedimentation, which represents hypostasis (a normal postmortem change), appears on PMCT as a gravity-dependent separation into two distinct layers: a high-density dependent layer and a low-density non-dependent layer, with a horizontal interface between both layers [3]. Recent forensic studies have demonstrated that specific high-density patterns in cardiac chambers on PMCT strongly suggest the presence of intracardiac blood clots at autopsy [4]. A previous pilot study demonstrated associations between blood sedimentation and antemortem fibrinogen levels [5]; however, the relationships between postmortem blood clots and patients’ antemortem clinical parameters remain unexplored.

Interpreting postmortem blood clots is challenging, especially in cases of in-hospital deaths, where patients often have complex medical conditions and receive treatments affecting blood coagulation [6–8]. In forensic medicine, recent studies have demonstrated that various clinical factors, including inflammatory conditions involving neutrophil extracellular traps (NETs), might influence postmortem clot formation [9, 10]. Despite the frequency of blood clots observed on PMCT, their relationship with antemortem clinical conditions is yet to be systematically investigated.

Previous research has described postmortem intracardiac/intravascular changes in PMCT; however, several critical knowledge gaps persist. First, PMCT commonly reveals two distinct patterns of blood distribution: clot formation and sedimentation. However, the relationship between antemortem clinical conditions and the development of these patterns is yet to be systematically investigated. Second, while a pilot study has shown associations between blood sedimentation and fibrinogen levels [5], the broader spectrum of clinical and laboratory parameters that might influence these postmortem findings remains unknown. Third, the potential predictive value of these postmortem findings for understanding the patient's antemortem clinical status is yet to be explored. Understanding these relationships is crucial for forensic and clinical interpretation of patient’s PMCT findings.

Therefore, in this study, we examined the associations between cardiac and vascular PMCT findings, blood clots and sedimentation, and patients' antemortem clinical and laboratory parameters in cases of in-hospital death.

## Material and methods

### Study design and ethical considerations

The Institutional Review Board of the University of Tokyo Hospital approved this prospective observational study (Ethical Committee No. 2076, June 9, 2008). The study protocol was designed and implemented in accordance with the ethical principles of the Declaration of Helsinki. Written informed consent was prospectively obtained from each deceased patient's next of kin before conducting PMCT examinations and pathological autopsies. All participants' next of kin were given a detailed explanation of the study's purpose, procedures, and the use of data for research purposes, and informed of their right to withdraw consent at any time. In addition, measures to protect participant confidentiality were explained.

PMCT examinations and autopsy were conducted at the University of XXX Hospital between April 2009 and December 2018. The study protocol was established before initiating data collection, with predefined inclusion and exclusion criteria, standardized imaging protocols, and systematic data collection procedures.

### Case selection

**Fig. 1:**
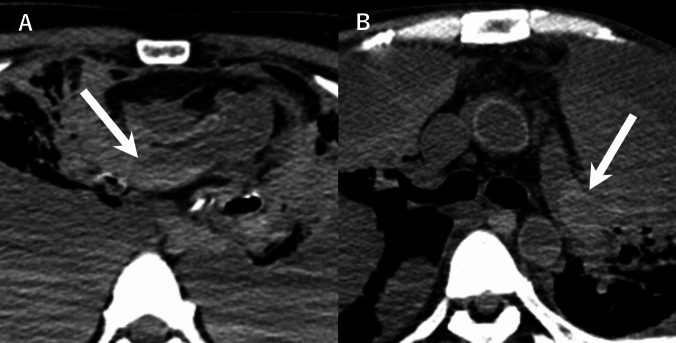
Examples of pulmonary arterial thrombosis diagnosed by autopsy. **A** 74-year-old female. Axial postmortem CT image showing dependent high-density area in the right pulmonary artery (arrow) that resembles gravitational sedimentation as a postmortem change. CT was performed 488 min after death. **B** 45-year-old male. Axial postmortem CT image showing high-density area along the vessel wall in the left pulmonary artery (arrow) that resembles cast-like blood clot as a postmortem change. CT was performed 128 min after death

We included cases that met the following criteria: (a) adult patients aged ≥ 18 years, (b) complete blood tests performed within 1 week before death, and (c) PMCT conducted within 24 h after death. We excluded cases with compromised CT image quality that prevented accurate assessment and cases that could interfere with the accurate evaluation of postmortem blood distribution patterns. These include (a) presence of cardiovascular devices that could affect blood flow patterns (such as left ventricular assist devices or cardiac pacing devices), (b) suspected conditions causing major blood loss or displacement (such as massive intra-thoracic or intra-abdominal hemorrhage, thoracic aortic dissection, or ruptured thoracic aortic aneurysm on PMCT), (c) extensive intravascular gas that could obscure blood distribution patterns (defined as gas occupying ≥ 50% of the lumen cross-sectional area in any axial CT image of the cardiac cavities, pulmonary artery, or thoracic aorta), and (d) thrombi confirmed during autopsy, as it is difficult to differentiate between postmortem blood clots and antemortem thrombi on PMCT images (Fig. 1). Antemortem thrombi, confirmed during autopsy through histopathological examination showing organization and adherence to vessel walls, were distinguished from postmortem blood clots, which lack these features [11, 12]. Cases with confirmed antemortem thrombi were excluded to ensure that only postmortem blood distribution patterns were analyzed.

### Clinical and laboratory data collection

Two board-certified radiologists (with 10 and 14 years of experience) independently obtained patients’ clinical parameters from electronic medical records, and any discrepancies were resolved by consensus. The collected parameters include age, sex, cardiopulmonary resuscitation status, time between death and PMCT, presence of active malignancies (solid and hematologic malignancies), and antiplatelet/anticoagulant medications.

We evaluated the presence of pneumonia (infectious pneumonia) and non-pneumonic infections, focusing on pneumonia due to its high mortality rate among hospitalized patients [13, 14]. Non-pneumonic infections included urinary tract infections, bloodstream infections, surgical site infection (e.g., superficial abdominal wall infection), hepatobiliary infections (e.g., liver abscess, cholangitis), and soft tissue infections (e.g., cellulitis). Pneumonia diagnosis was based on the clinical symptoms (fever, cough, dyspnea, etc.), physical examination findings, laboratory data (elevated inflammatory markers, positive sputum cultures), and radiological findings documented in the patient’s medical records. Non-pneumonic infections were defined as documented infections with positive cultures from any site except respiratory specimens, or clinical diagnoses of infection with documented antimicrobial treatment during the 14-d period before death. Blood culture results from 14 days before death were also recorded.

The laboratory data obtained within 1 week before death include red blood cell count (RBC), hemoglobin (Hb), hematocrit (Ht), white blood cell count, neutrophil percentage, platelet count (PLT), blood urea nitrogen (BUN), creatinine (Cr), C-reactive protein, prothrombin time-international normalized ratio (PT-INR), activated partial thromboplastin time (APTT), D-dimer, and fibrinogen. We also noted cases with missing laboratory data. Medication use was determined based on medical record review; unclear cases were recorded as missing data.

### PMCT technique

All cadavers were maintained in the supine position at hospital room temperature until PMCT completion. PMCT was performed using either ROBUSTO (Hitachi Medical Corporation, Tokyo, Japan) or Aquilion (Canon Medical Systems Corporation, Otawara, Japan) multidetector CT scanners. The scanning parameters were helical mode, 2.5-mm slice thickness, 1.25-mm slice interval, 0.5-s rotation time, 120 kVp, and 250 mA. Images were reconstructed at 5-mm thickness with a 350-mm field of view and 512 × 512 matrix. No contrast medium was administered.

### Image analysis

**Fig. 2:**
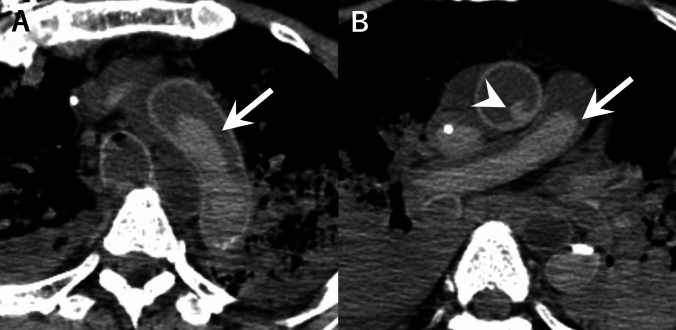
Example of a blood clot. A 64-year-old man with pancreatic carcinoma. **A** Axial postmortem CT showing cast-like blood clots (arrow) in the aortic arch. **B** Axial postmortem CT showing cast-like blood clot (arrow) in the pulmonary artery and ascending aorta (arrowhead). CT was performed 224 min after death

**Fig. 3:**
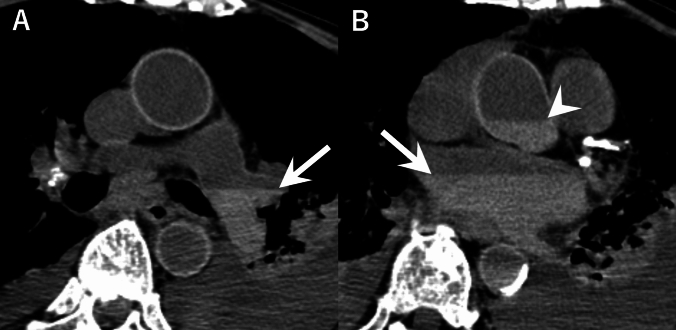
Example of gravitational sedimentation. An 81-year-old woman with acute myeloid leukemia. **A** Axial postmortem CT showing gravitational sedimentation (arrow) in the pulmonary artery. **B** Axial postmortem CT showing gravitational sedimentation in the left atrium (arrow) and ascending aorta (arrowhead). CT was performed 155 min after death

**Fig. 4:**
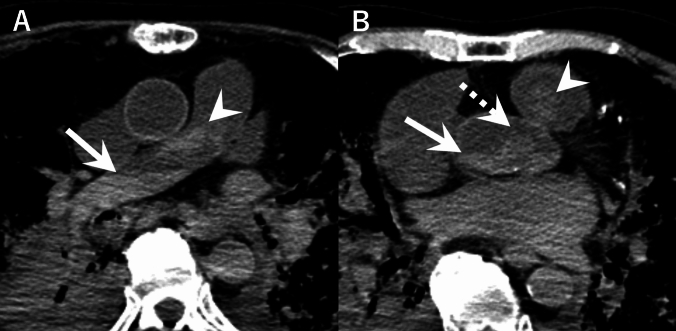
Example of uncertain differentiation between a blood clot and gravitational sedimentation. A 79-year-old man with interstitial pneumonia. **A** Axial postmortem CT showing a mixed pattern of gravitational sedimentation (arrow) and clot-like high-density area (arrowhead) in the pulmonary artery. **B** Axial postmortem CT showing gravitational sedimentation (arrow) and clot-like high-density areas in the ascending aorta (dotted arrow) and pulmonary artery (arrowhead). CT was performed 482 min after death

Two board-certified radiologists (with 13 and 16 years of PMCT experience) independently evaluated the PMCT images using RadiAnt DICOM Viewer (Medixant, Poznan, Poland). The images were viewed using a window width of 250 HU and a window level of 40 HU. Radiologists were able to adjust the window settings of the viewer as needed. With reference to previous PMCT study [4], the radiologists classified intracardiac/intravascular high-density areas in the right atrium (RA), left atrium (LA), pulmonary artery (PA), and thoracic aorta (TA) into one of four categories: (a) blood clot (high-density, cast-like structures; Fig. 2), (b) gravitational sedimentation (separation of blood into two distinct layers: a high-density dependent layer and a low-density non-dependent layer, with a horizontal boundary between them; Fig. 3), (c) uncertain differentiation between blood clot and gravitational sedimentation (Fig. 4), or (d) no high-density area.

### Autopsy technique

Conventional autopsies, including investigations of the thrombosis in the pulmonary artery, thoracic aorta and cardiac cavities, were performed by two board-certified pathologists immediately after PMCT (within 1 h) in all cases. The pathologists were informed of the patient’s clinical histories but were blinded to the radiologists’ PMCT reports. Gross inspection and histological analyses were performed for each organ using standardized methods. Further detailed histological examinations were conducted on necessary organs at the pathologist's discretion. The cause of death was determined or estimated when possible.

### Statistical analysis

Statistical analyses were performed using IBM SPSS Statistics for Windows version 29.0 (IBM Corp., Armonk, NY, USA). Interrater agreement was assessed using Cohen's kappa coefficient. For each anatomical location (RA, LA, PA, and TA), comparisons between blood clot and blood sedimentation groups (categories a and b, respectively) were conducted using the Mann–Whitney U test for continuous variables (age, time between death and PMCT, and all laboratory parameters) and chi-square test for categorical variables (sex, cardiopulmonary resuscitation status, pneumonia, other infections, blood culture results, antiplatelet/anticoagulant medications, and malignancies), excluding cases with uncertain differentiation or no high-density areas (categories c and d, respectively). Patients who presented with both pneumonia and non-pneumonic infections were recorded as positive for both variables and included in both respective statistical analyses. This approach was chosen to evaluate the independent effects of each infection type on postmortem blood distribution patterns. A *p*-value < 0.05 was considered statistically significant for all analyses. Univariate logistic regression analysis was performed for clinical and laboratory parameters to investigate factors associated with the formation of blood clots versus gravitational sedimentation. For logistic regression analyses, the blood clot and sedimentation groups were coded as 0 (reference) and 1, respectively, implying that odds ratios (ORs) > 1 indicate positive associations with sedimentation formation. In contrast, OR < 1 indicates positive associations with clot formation. The variables for multivariate logistic regression analysis were selected based on their statistical significance in the univariate analysis (*p* < .05) and clinical importance while considering multicollinearity.

## Results

### Study population

**Fig. 5:**
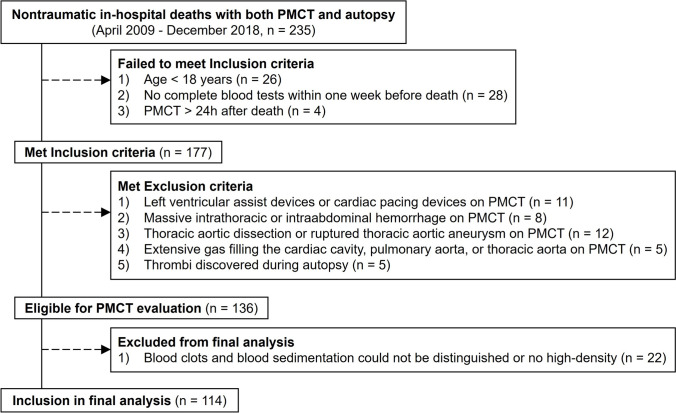
Flow diagram showing the case selection process

During the study period, both PMCT and autopsy were conducted for 235 consecutive non-traumatic in-hospital deaths. After applying the inclusion and exclusion criteria, 136 cases were eligible for CT evaluation (Fig. 5). Of these, 22 cases were excluded during the CT image analysis with either uncertain differentiation between blood clot and gravitational sedimentation or no high density in any area. The final study population comprised 114 cases: 78 males (68%) and 36 females (32%), with a mean age of 68.0 ± 12.3 years. Among these cases, 48 (42%) had solid malignancies, 23 (20%) had hematologic malignancies, 41 (36%) had pneumonia, and 25 (22%) had non-pneumonic infections. Positive blood cultures were observed in 24 cases (21%). Antiplatelet medication was not used in any cases, and anticoagulant medication was used in 2 cases (2%). The assessment of blood distribution patterns in these 114 cases demonstrated excellent inter-observer agreement for all anatomical sites (κ = 0.92 for RA, 0.87 for LA, 0.88 for PA, and 0.91 for TA).

### Distribution of PMCT findings

Blood clots and blood sedimentation showed distinct patterns across the anatomical sites. In the RA, blood clots and blood sedimentation were present in 60 (53%) and 48 (42%) cases, respectively. In the LA, blood clots and blood sedimentation were observed in 57 (50%) and 53 cases (46%), respectively. In the PA, blood clots were present in 59 cases (52%) and blood sedimentation in 39 cases (34%). The TA showed blood clots in 60 cases (53%) and blood sedimentation in 47 cases (41%).

### Clinical and laboratory characteristics

In the blood sedimentation group, non-pneumonic infections and blood culture positivity were significantly more frequent across all anatomical sites (all *p* < .05). Solid malignancy was significantly more common in the blood clot group (all *p* < .05) (Table 1).

**Table 1:** Clinical characteristics of the blood clot and sedimentation groups

Characteristics	Location	No. of cases (C/S [missing data])	Clot group	Sedimentation group	p value
Age (years)	RA	60/48	68.7 ± 11.9	68.1 ± 12.2	.55
	LA	57/53	68.4 ± 11.2	68.8 ± 12.1	> .99
	PA	59/39	68.2 ± 11.8	68.7 ± 13.1	.96
	TA	60/47	68.8 ± 11.7	68.0 ± 12.2	.59
Sex (male/female)	RA	60/48	35 (58%)/25	38 (79%)/10	.024*
	LA	57/53	35 (61%)/22	39 (74%)/14	.22
	PA	59/39	33 (56%)/26	29 (74%)/10	.09
	TA	60/47	37 (62%)/23	35 (76%)/12	.21
Cardiopulmonary resuscitation (yes/no)	RA	60/48	3 (5%)/57	1 (2%)/47	.63
	LA	57/53	3 (5%)/54	2 (4%)/51	> .99
	PA	59/39	2 (3%)/57	0 (0%)/39	.52
	TA	60/47	3 (5%)/57	2 (4%)/45	> .99
PMCT interval (min)	RA	60/48	536.3 ± 383.1	571.8 ± 381.7	.49
	LA	57/53	554.2 ± 393.8	542.8 ± 326.4	.87
	PA	59/39	541.7 ± 387.5	563.2 ± 338.3	.55
	TA	60/47	528.2 ± 374.7	544.7 ± 423.1	.97
Solid malignancy	RA	60/48	34 (57%)	14 (29%)	.006*
	LA	57/53	34 (60%)	14 (26%)	< .001*
	PA	59/39	33 (56%)	12 (31%)	.022*
	TA	60/47	33 (55%)	13 (28%)	.006*
Hematologic malignancy	RA	60/48	9 (15%)	13 (27%)	.15
	LA	57/53	7 (12%)	16 (30%)	.033*
	PA	59/39	9 (15%)	10 (26%)	.30
	TA	60/47	9 (15%)	14 (30%)	.10
Pneumonia	RA	60/48	23 (38%)	18 (38%)	> .99
	LA	57/53	22 (39%)	18 (34%)	.69
	PA	59/39	22 (37%)	14 (36%)	> .99
	TA	60/47	25 (42%)	15 (32%)	.32
Non-pneumonic infections	RA	53/47 (7/1)	5 (9%)	19 (40%)	< .001*
	LA	52/52 (5/1)	7 (14%)	18 (35%)	.021*
	PA	52/37 (7/2)	6 (12%)	17 (46%)	< .001*
	TA	54/44 (6/3)	7 (13%)	17 (39%)	.004*
Positive blood culture	RA	55/48 (5/0)	7 (13%)	17 (35%)	.010*
	LA	53/52 (4/1)	6 (12%)	17 (32%)	.017*
	PA	53/39 (6/0)	6 (11%)	14 (36%)	.009*
	TA	55/46 (5/1)	8 (15%)	15 (33%)	.035*
Antiplatelet use	RA	56/48 (4/0)	0 (0%)	0 (0%)	NA
	LA	53/53 (4/0)	0 (0%)	0 (0%)	NA
	PA	55/39 (4/0)	0 (0%)	0 (0%)	NA
	TA	56/47 (4/0)	0 (0%)	0 (0%)	NA
Anticoagulant use	RA	56/48 (4/0)	1 (2%)	1 (2%)	> .99
	LA	53/52 (4/1)	1 (2%)	1 (2%)	> .99
	PA	55/38 (4/1)	1 (2%)	1 (3%)	> .99
	TA	56/46 (4/1)	1 (2%)	0 (0%)	> .99

In the column of “No. of Cases,” when present, numbers in parentheses indicate missing data

Data are presented as mean ± standard deviation for continuous variables and number (percentage) for categorical variables

For categorical variables with missing data, percentages were calculated after excluding missing cases

*RA* right atrium, *LA* left atrium, *PA* pulmonary artery, *TA* thoracic aorta, *C* clot, *S* sedimentation, *PMCT* postmortem computed tomography, *NA* not applicable

*Statistically significant (*p* < .05)

Laboratory parameters showed consistent differences between groups. The blood clot group demonstrated significantly higher values for hematologic parameters including PLT, RBCs, Hb, and neutrophil percentage (all *p* < .05). Conversely, the blood sedimentation group showed significantly higher values for coagulation parameters including PT-INR and APTT (all *p* < .05). Fibrinogen levels were lower in the sedimentation group, with statistical significance in the PA (*p* = .033) and TA (*p* = .027) (Table 2).

**Table 2:** Laboratory parameters in the blood clot and sedimentation groups

Parameters	Location	No. of cases (C/S [missing data])	Clot group	Sedimentation group	p value
*Hematologic parameters*					
Red blood cells (× 10⁶/μL)	RA	60/48	322.2 ± 86.3	273.6 ± 64.0	.002*
	LA	57/53	323.0 ± 86.9	273.9 ± 66.6	.004*
	PA	59/39	321.9 ± 86.2	276.8 ± 57.7	.006*
	TA	60/47	321.5 ± 85.2	274.8 ± 64.9	.004*
Hemoglobin (g/dL)	RA	60/48	9.7 ± 2.6	8.4 ± 2.3	.021*
	LA	57/53	9.8 ± 2.5	8.4 ± 2.3	.010*
	PA	59/39	9.8 ± 2.5	8.6 ± 2.1	.029*
	TA	60/47	9.8 ± 2.5	8.4 ± 2.3	.009*
Hematocrit (%)	RA	60/48	29.7 ± 8.5	26.1 ± 6.1	.006*
	LA	57/53	29.8 ± 8.5	26.0 ± 6.3	.007*
	PA	59/39	29.6 ± 8.5	26.6 ± 5.5	.022*
	TA	60/47	29.8 ± 8.3	26.0 ± 6.2	.004*
White blood cells (× 10^3^/μL)	RA	60/48	13.8 ± 11.2	17.5 ± 33.7	.21
	LA	57/53	14.2 ± 11.3	15.3 ± 31.6	.043*
	PA	59/39	13.7 ± 11.3	17.5 ± 36.4	.21
	TA	60/47	14.5 ± 12.9	16.0 ± 33.4	.08
Neutrophil (%)	RA	58/44 (2/4)	82.0 ± 20.4	69.9 ± 30.3	.012*
	LA	55/49 (2/4)	82.3 ± 20.5	69.3 ± 29.4	.004*
	PA	57/37 (2/2)	83.6 ± 17.7	68.9 ± 32.1	.009*
	TA	58/43 (2/4)	81.7 ± 20.3	68.3 ± 31.0	.013*
Platelets (× 10^3^/μL)	RA	60/48	20.8 ± 15.0	8.3 ± 7.7	< .001*
	LA	57/53	21.0 ± 15.3	8.5 ± 7.8	< .001*
	PA	59/39	20.7 ± 15.3	7.9 ± 7.6	< .001*
	TA	60/47	20.9 ± 14.9	7.9 ± 7.5	< .001*
*Chemistry parameters*					
BUN (mg/dL)	RA	60/48	51.8 ± 42.2	66.7 ± 45.2	.042*
	LA	57/53	49.2 ± 39.7	73.1 ± 53.4	.011*
	PA	59/39	50.0 ± 37.1	72.1 ± 54.4	.031*
	TA	60/47	48.9 ± 39.2	73.1 ± 53.4	.010*
Creatinine (mg/dL)	RA	60/48	1.9 ± 2.2	2.6 ± 2.2	.004*
	LA	57/53	1.6 ± 1.6	2.9 ± 2.7	.001*
	PA	59/39	1.7 ± 1.8	2.7 ± 2.3	.004*
	TA	60/47	1.6 ± 1.6	2.9 ± 2.4	< .001*
C-reactive Protein (mg/dL)	RA	60/48	12.7 ± 12.0	11.6 ± 11.2	.67
	LA	57/53	12.3 ± 11.6	11.6 ± 11.6	.61
	PA	59/39	12.3 ± 11.9	9.8 ± 7.3	.50
	TA	60/47	12.9 ± 11.9	10.9 ± 11.4	.28
*Coagulation parameters*					
PT-INR	RA	52/45 (8/3)	1.3 ± 0.4	1.8 ± 1.1	< .001*
	LA	48/50 (9/3)	1.3 ± 0.6	1.7 ± 1.0	.004*
	PA	51/38 (8/1)	1.3 ± 0.5	1.7 ± 1.0	.004*
	TA	52/44 (9/3)	1.3 ± 0.6	1.8 ± 1.0	.002*
APTT (seconds)	RA	51/45 (9/3)	37.4 ± 21.2	54.9 ± 39.8	< .001*
	LA	47/50 (10/3)	37.0 ± 20.2	54.3 ± 38.7	< .001*
	PA	50/38 (9/1)	36.0 ± 19.1	50.9 ± 28.9	.001*
	TA	51/44 (10/3)	36.5 ± 19.6	55.4 ± 40.0	< .001*
d-Dimer (μg/mL)	RA	40/33 (20/15)	12.3 ± 19.6	32.3 ± 66.6	.15
	LA	38/35 (19/18)	13.1 ± 19.9	30.3 ± 65.1	.61
	PA	40/28 (19/11)	12.4 ± 19.6	36.7 ± 71.6	.07
	TA	40/32 (21/15)	11.9 ± 19.3	32.7 ± 67.7	.26
Fibrinogen (mg/dL)	RA	48/36 (12/12)	390.1 ± 182.2	313.2 ± 165.4	.08
	LA	44/41 (13/12)	386.9 ± 181.2	312.0 ± 167.3	.08
	PA	47/30 (12/9)	388.6 ± 186.8	288.4 ± 146.9	.033*
	TA	47/36 (14/11)	399.2 ± 180.0	302.2 ± 160.7	.027*

In the “No. of Cases” column, when present, numbers in parentheses indicate missing data. Data are presented as mean ± standard deviation for continuous variables. For continuous variables with missing data, percentages were calculated after excluding missing cases

*C* clot, *S* sedimentation, *BUN* blood urea nitrogen, *CRP* C-reactive protein, *PT*-*INR* prothrombin time-international normalized ratio, *APTT* activated partial thromboplastin time

*Statistically significant (*p* < .05)

### Predictive factors

Univariate analysis revealed that non-pneumonic infections and blood culture positivity were strongly associated with blood sedimentation across all sites. Solid malignancy showed a significant association with blood clot formation. Of the laboratory parameters, lower PLT, Hb levels, and neutrophil percentages were associated with blood sedimentation. Elevated Cr levels showed significant associations with blood sedimentation in the PA and TA, and a borderline significant association in the LA. Regarding coagulation parameters, elevated APTT showed significant associations with blood sedimentation across all sites, while elevated PT-INR showed significant associations in the RA, PA, and TA, with a borderline significant association in the LA. Lower fibrinogen levels are significantly associated with blood sedimentation in the PA and TA. BUN levels are significantly associated with blood sedimentation in the LA, PA, and TA.

The final multivariate model included 10 variables: sex, solid malignancy, non-pneumonic infection, Hb, neutrophil percentage, PLT, Cr, PT-INR, APTT, and fibrinogen. In the multivariate analysis, non-pneumonic infections maintained strong associations with blood sedimentation across all sites. Higher PLTs independently predicted blood clot formation in the RA, LA, and TA. Although fibrinogen levels showed statistically significant p-values in the analyses of the PA and TA, ORs of approximately 1.0 indicated little meaningful association with either blood clot formation or blood sedimentation. Other laboratory parameters also showed no significant associations in the multivariate analysis (Table 3).

**Table 3:** Univariate and multivariate analyses for predicting blood sedimentation

Variable	Location	Univariate analysis	Multivariate analysis
*Clinical factors*			
Age	RA	.80, 1.00 (0.97–1.03)	–
	LA	.87, 1.00 (0.97–1.03)	–
	PA	.85, 1.00 (0.97–1.04)	–
	TA	.75, 1.00 (0.96–1.03)	–
Sex	RA	.024^†^, 0.37 (0.15–0.88)	.09, 0.18 (0.03–1.29)
	LA	.18, 0.57 (0.25–1.29)	.81, 0.83 (0.19–3.64)
	PA	.07, 0.44 (0.18–1.06)	.29, 0.34 (0.05–2.53)
	TA	.16, 0.55 (0.24–1.27)	.72, 0.73 (0.13–4.03)
Cardiopulmonary resuscitation	RA	.44, 0.40 (0.04–4.02)	–
	LA	.71, 0.71 (0.11–4.40)	–
	PA	> .99, 0.00 (0.00)	–
	TA	.86, 0.84 (0.14–5.27)	–
PMCT interval	RA	.63, 1.00 (1.00)	–
	LA	.87, 1.00 (1.00)	–
	PA	.78, 1.00 (1.00)	–
	TA	.83, 1.00 (1.00)	–
Solid malignancy	RA	.005^†^, 0.32 (0.14–0.70)	.69, 1.45 (0.23–9.24)
	LA	< .001^†^, 0.24 (0.11–0.55)	.46, 0.57 (0.13–2.54)
	PA	.016^†^, 0.35 (0.15–0.82)	.77, 1.35 (0.17–10.61)
	TA	.005^†^, 0.31 (0.14–0.71)	.94, 0.93 (0.15–5.91)
Hematologic malignancy	RA	.13, 2.11 (0.81–5.16)	–
	LA	.025^†^, 3.09 (1.15–8.27)	–
	PA	.21, 1.92 (0.70–5.26)	–
	TA	.07, 2.40 (0.93–6.19)	–
Pneumonia	RA	.93, 0.97 (0.44–2.11)	–
	LA	.61, 0.82 (0.38–1.78)	–
	PA	.89, 0.94 (0.41–2.18)	–
	TA	.27, 0.64 (0.29–1.42)	–
Non-pneumonic infection*	RA	< .001^†^, 6.51 (2.19–19.37)	.005^†^, 116.23 (4.19–3226.65)
	LA	.014^†^, 3.40 (1.23–9.07)	.013^†^, 12.51 (1.69–92.40)
	PA	< .001^†^, 6.52 (2.24–18.97)	.006^†^, 114.50 (3.87–3387.12)
	TA	.005^†^, 4.23 (1.56–11.49)	.006^†^, 36.61 (2.87–466.70)
Positive blood culture	RA	.009^†^, 3.76 (1.40–10.11)	–
	LA	.014^†^, 3.62 (1.30–10.12)	–
	PA	.007^†^, 4.39 (1.50–12.82)	–
	TA	.035^†^, 2.84 (1.08–7.50)	–
Anticoagulant use*	RA	.91, 1.17 (0.07–19.22)	–
	LA	.99, 1.02 (0.06–16.74)	–
	PA	.79, 1.46 (0.09–24.08)	–
	TA	> .99, 0.00 (0.00)	–
*Laboratory parameters*			
Red blood cells	RA	.003^†^, 0.99 (0.99–1.00)	–
	LA	.002^†^, 0.99 (0.99–1.00)	–
	PA	.008^†^, 0.99 (0.99–1.00)	–
	TA	.004^†^, 0.99 (0.99–1.00)	–
Hemoglobin	RA	.016^†^, 0.81 (0.69–0.96)	.46, 0.85 (0.55–1.31)
	LA	.005^†^, 0.78 (0.65–0.93)	.90, 1.02 (0.73–1.43)
	PA	.023^†^, 0.81 (0.66–0.97)	.79, 0.94 (0.60–1.48)
	TA	.007^†^, 0.78 (0.65–0.93)	.82, 1.04 (0.93–1.49)
Hematocrit	RA	.020^†^, 0.94 (0.89–0.99)	–
	LA	.013^†^, 0.93 (0.89–0.99)	–
	PA	.06, 0.95 (0.89–1.00)	–
	TA	.014^†^, 0.93 (0.88–0.99)	–
White blood cells	RA	.46, 1.01 (0.99–1.02)	–
	LA	.80, 1.00 (0.99–1.02)	–
	PA	.48, 1.01 (0.99–1.02)	–
	TA	.76, 1.00 (0.99–1.02)	–
Neutrophil*	RA	.025^†^, 0.98 (0.96–1.00)	.49, 0.99 (0.96–1.02)
	LA	.016^†^, 0.98 (0.96–1.00)	.15, 0.98 (0.96–1.01)
	PA	.011^†^, 0.98 (0.96–1.00)	.17, 0.98 (0.94–1.01)
	TA	.016^†^, 0.98 (0.96–1.00)	.12, 0.98 (0.95–1.01)
Platelets	RA	< .001^†^, 0.90 (0.86–0.94)	.016^†^, 0.86 (0.76–0.97)
	LA	< .001^†^, 0.90 (0.86–0.95)	.012^†^, 0.90 (0.82–0.98)
	PA	< .001^†^, 0.90 (0.85–0.95)	.08, 0.88 (0.77–1.01)
	TA	< .001^†^, 0.89 (0.85–0.94)	.034^†^, 0.89 (0.79–0.99)
BUN	RA	.085, 1.01 (0.99–1.02)	–
	LA	.012^†^, 1.01 (1.00–1.02)	–
	PA	.025^†^, 1.01 (1.00–1.02)	–
	TA	.012^†^, 1.01 (1.00–1.02)	–
Creatinine	RA	.092, 1.17 (0.98–1.41)	.33, 0.84 (0.59–1.20)
	LA	.005^†^, 1.36 (1.10–1.69)	.78, 1.31 (0.94–1.77)
	PA	.026^†^, 1.27 (1.03–1.56)	.73, 1.08 (0.69–1.70)
	TA	.003^†^, 1.41 (1.13–1.76)	.098, 1.34 (0.95–1.90)
C-reactive protein	RA	.62, 0.99 (0.96–1.03)	–
	LA	.74, 1.00 (0.96–1.03)	–
	PA	.25, 0.97 (0.93–1.02)	–
	TA	.40, 0.99 (0.95–1.02)	–
PT-INR*	RA	.006^†^, 3.48 (1.44–8.41)	.68, 1.32 (0.36–4.86)
	LA	.05, 1.94 (1.00–3.75)	.31, 0.61 (0.23–1.60)
	PA	.016^†^, 2.63 (1.20–5.77)	.93, 1.06 (0.28–3.95)
	TA	.030^†^, 2.10 (1.07–4.10)	.33, 0.61 (0.23–1.67)
APTT*	RA	.018^†^, 1.02 (1.00–1.05)	.81, 1.00 (0.97–1.04)
	LA	.016^†^, 1.03 (1.01–1.05)	.46, 1.01 (0.98–1.04)
	PA	.014^†^, 1.03 (1.01–1.06)	.93, 1.00 (0.99–1.00)
	TA	.011^†^, 1.03 (1.01–1.06)	.86, 0.99 (0.99–1.00)
d-Dimer*	RA	.14, 1.01 (1.00–1.03)	–
	LA	.18, 1.01 (1.00–1.02)	–
	PA	.11, 1.01 (1.00–1.03)	–
	TA	.14, 1.01 (1.00–1.03)	–
Fibrinogen*	RA	.05, 1.00 (1.00)	.10, 1.00 (0.99–1.00)
	LA	.06, 1.00 (1.00)	.35, 1.0 (0.99–1.00)
	PA	.019^†^, 1.00 (0.99–1.00)	.026^†^, 0.99 (0.99–1.00)
	TA	.017^†^, 1.00 (0.99–1.00)	.022^†^, 0.99 (0.99–1.00)

Each cell shows a *p*-value followed by an odds ratio (95% confidence interval). Antiplatelet medication use was not analyzed due to insufficient distribution of cases for meaningful statistical comparison. Odds ratios > 1 indicate association with blood clot formation, odds ratio = 1 indicates no association, and odds ratios < 1 indicate association with blood sedimentation. Dash (–) indicates variables not included in multivariate analysis

*RA* right atrium, *LA* left atrium, *PA* pulmonary artery, *TA* thoracic aorta, *PMCT* postmortem computed tomography, *BUN* blood urea nitrogen, *PT*-*INR* prothrombin time-international normalized ratio, *APTT* activated partial thromboplastin time

*Variables with missing data were analyzed after excluding missing cases

^†^Statistically significant (*p* < .05)

## Discussion

This study’s findings revealed that postmortem blood sedimentation was associated with non-pneumonic infections and that blood clot formation was associated with higher PLTs on CT across cardiac and vascular locations.

Systemic infections or sepsis have been reported to often pronounce blood coagulation and lead to disseminated intravascular coagulation and consumption coagulopathy [15]. The present study's strong association between non-pneumonic infections and blood sedimentation is supposed to have reflected the infection-related inflammatory responses on blood coagulation. The present study’s higher frequency of blood culture positivity and elevated coagulation parameters in the blood sedimentation group also corresponds to the above-mentioned knowledge.

Neutrophils reportedly have a crucial role in clot formation through NETs, where neutrophils release extracellular deoxyribonucleic acid in response to inflammatory stimuli [9, 10]. This process, known as NETosis, is a distinct form of programmed cell death where neutrophils expel their deoxyribonucleic acid and associated proteins to form web-like structures that contribute to clot formation. The "chicken fat clots" observed at autopsies, which correspond to the blood clots seen on PMCT, have been immunohistochemically proven to contain proteins associated with NET formation [16, 17]. In the univariate analysis in the present study, the blood clot group showed higher neutrophil percentages than the sedimentation group; however, this association did not persist in the multivariate analysis, suggesting other factors, particularly non-pneumonic infections may mediate this relationship.

The higher RBCs, Hb, and Ht values in the blood clot group suggest important relationships between blood cellular components and postmortem clot formation, which aligns with the known role of RBCs in clot formation [18]. Higher concentrations of RBCs may promote clot formation through multiple mechanisms [19]: increased blood viscosity can slow blood flow in the agonal phase, and higher RBC concentrations may facilitate platelet-vessel wall interactions through platelet margination [18, 20].

In the univariate analysis, solid malignancies were associated with blood clot formation, aligning with the well-documented hypercoagulable state in patients with cancer [21, 22]. Similarly, in the univariate analysis, elevated Cr levels showed associations with blood sedimentation in some anatomical locations, potentially reflecting the complex effects of renal dysfunction on coagulation through mechanisms such as platelet dysfunction and endothelial damage. However, neither of these associations persisted in the multivariate analysis when adjusted for factors such as PLT and infection status, providing evidence of a complex pathophysiological interplay.

Associations between elevated antemortem fibrinogen levels and blood sedimentation on PMCT have been previously reported [5]. Although the fibrinogen levels showed statistical significance in the analyses of the PA and TA, the effect size was too small to support a meaningful association between fibrinogen levels and postmortem blood distribution patterns. This suggests that the relationship between fibrinogen levels and postmortem blood appearances may be more complex than previously reported [5].

The associations between non-pneumonic infections and PLTs with postmortem intracardiac/intravascular CT findings were consistent across most examined locations, while some factors like fibrinogen showed location-specific associations. This consistency suggests that these particular factors influence blood clot formation and sedimentation throughout the major vessels and cardiac chambers rather than having location-specific effects. While previous studies have primarily focused on forensic applications [4], our findings extend the utility of PMCT blood distribution patterns to clinical settings, potentially providing valuable information about a patient's antemortem status, particularly in cases with limited clinical information.

The above-mentioned findings in the present study have important implications for forensic and clinical practice. First, they provide objective criteria for interpreting postmortem intracardiac/intravascular CT findings by establishing clear associations with antemortem clinical parameters. Second, blood gravitational sedimentation on PMCT might indicate antemortem inflammatory status, potentially providing valuable information in cases where the patient’s clinical history is limited. Third, the consistent relationship with PLTs offers a new perspective on the mechanisms of postmortem blood alterations.

Our study has some limitations. As an initial single-center prospective investigation, our findings may not be generalizable to other institutions or populations. While all laboratory tests were performed within 1 week before death, the varying timing of these tests relative to death may have influenced our results. Blood status is dependent on the overall patient condition. Caution should be exercised when considering whether the local assessment of the heart and major blood vessels conducted in this study accurately reflects the systemic condition. Another limitation is the lack of comprehensive data on perimortem fluid therapy, which could potentially affect the intravascular findings. While fluid administration orders could be extracted from electronic medical records, documentation was inconsistent, with some cases having incomplete records of actual administered volumes, particularly during emergency situations. Our relatively small sample size and missing laboratory data limited the study’s statistical power, particularly for subgroup analyses. Finally, excluding cases with extensive postmortem gas formation or diagnosed thrombi may have introduced selection bias.

## Conclusion

Postmortem gravitational sedimentation was associated with non-pneumonic infections, and clot formation was associated with higher PLTs on CT across cardiac and vascular locations. These findings provide objective criteria for interpreting postmortem intracardiac and intravascular CT appearances, which may enhance our ability to understand a patient's antemortem clinical status during forensic and clinical evaluations.

## Data Availability

The data that support the findings of this study are available from the corresponding author upon reasonable request.
